# Cas fatal de co-infection par le virus de la fièvre jaune et le SARS-CoV2 pendant la pandémie de Covid-19 de 2020 en Guyane

**DOI:** 10.48327/mtsi.v4i3.2024.445

**Published:** 2024-09-12

**Authors:** Caroline THOMAS, Clara PICHARD, Dominique ROUSSET, Magalie DEMAR, Félix DJOSSOU, Alice SANNA, Lise DUDOGNON, Mathieu NACHER, Jean-Marc PUJO, Céline MICHAUD, Mélanie GAILLET, Hatem KALLEL, Loïc EPELBOIN

**Affiliations:** 1 Laboratoire hospitalo-universitaire de parasitologie-mycologie, Centre hospitalier de Cayenne, Guyane. Service de réanimation polyvalente, Centre hospitalier universitaire (CHU) de Guadeloupe; 2 Unité des maladies infectieuses et tropicales, Centre hospitalier de Cayenne, Guyane; 3 Service de réanimation polyvalente du CHU de Guadeloupe, Les Abymes, Guadeloupe; 4 Équipe mobile de santé publique en communes (EMSPEC), Centre hospitalier de Cayenne, Guyane; 5 Laboratoire de virologie, Centre national de référence (CNR) arbovirus, Institut Pasteur de la Guyane, Cayenne, Guyane; 6 Agence régionale de santé de Guyane, Cayenne, Guyane; 7 Centre d'investigation clinique, Inserm 1424, CH de Cayenne, Guyane; 8 Service d'accueil des urgences et SAMU, CH de Cayenne, Guyane; 9 Centres délocalisés de prévention et de soins, CH de Cayenne, Guyane; 10 Service de médecine intensive réanimation, CH de Cayenne, Guyane

**Keywords:** *Orthoflavivirus flavi*, Fièvre jaune, SARS-CoV2, Vaccin, Décès, Co-infection, Kayodé, Guyane, Amérique du sud, *Orthoflavivirus flavi*, Yellow fever, SARS-CoV2, Vaccine, Death, Co-infection, Kayodé, French Guiana, South America

## Abstract

Le virus de la fièvre jaune (VFJ) récemment renommé *Orthoflavivirus flavi* est un arbovirus de la famille des Flaviviridae et du genre *Orthoflavivirus* endémique en Amérique du Sud et en Afrique tropicale. En Amérique latine, le Brésil a connu une épidémie d'une ampleur inégalée entre 2016 et 2018. La résurgence de nouveaux cas en Guyane ces dernières années a ravivé l'intérêt pour la maladie. En décembre 2019, la pandémie mondiale de Covid-19 a débuté et a rapidement atteint l'Amérique du Sud. Les premiers cas ont été recensés en Guyane en mars 2020. De nombreuses maladies tropicales circulent dans la région et augmentent ainsi la possibilité d'observer des co-infections. Nous rapportons ici le premier cas de co-infection VFJ-SARS-CoV2 chez un jeune Français amérindien de 14 ans vivant sur le Haut-Maroni, dont l'issue a été fatale en neuf jours. Il a reçu une dose unique de vaccin antiamaril dans l'enfance.

## Abstract (en)

The yellow fever virus (YFV), recently renamed *Orthoflavivirus flavi,* is an arbovirus of the Flaviviridae family and *Orthoflavivirus* genus endemic in South America and Tropical Africa. Brazil experienced an epidemic of unprecedented magnitude between 2016 and 2018. The resurgence of new cases in French Guiana in recent years has rekindled interest in the disease. In December 2019, the global pandemic of Covid-19 began and rapidly reached South America. The first cases were reported in French Guiana in March 2020. Many tropical diseases circulate in the region and the possibility of co-infections is therefore high. Here, we report the first case of YF virus-SARS-CoV2 co-infection in a 14-year-old French Amerindian boy who died within nine days. He had received a single dose of YF vaccine in childhood.

## Introduction

Le virus de la fièvre jaune (VFJ), récemment renommé *Orthoflavivirus flavi* est un arbovirus de la famille des Flaviviridae et du genre *Orthoflavivirus^1^* [3]. Il s'agit d'une arbovirose endémique, la plus grave qui circule en Amérique latine. En 2016-2018, une épidémie est survenue au Brésil, touchant des zones jusque-là indemnes [15]. Sur le plateau des Guyanes, qui inclut l’État de Bolivar au Venezuela, le Guyana, le Surinam, la Guyane et l’État de l'Amapá du Brésil, seuls neuf cas ont été rapportés entre 1990 et 2022 [22]. En décembre 2019, la pandémie mondiale de Covid-19 a débuté et a rapidement atteint l'Amérique du Sud. Les premiers cas ont été recensés en Guyane en mars 2020 [8]. La possibilité d'observer des coinfections est élevée dans cette zone où circulent de nombreux agents infectieux [6]. En effet, en juillet 2020, la Guyane connaît sa première vague de Covid-19 ainsi qu'une épidémie de dengue, principalement due au sérotype DENV2 [10, 19]. Sur le Haut-Maroni, il y a également régulièrement des cas sporadiques de paludisme, principalement à *Plasmodium vivax* [20]. Par ailleurs il y a toujours beaucoup de cas de leptospirose, qui participent à accroitre le nombre de diagnostics différentiels. Nous rapportons le cas d'un patient ayant présenté une co-infection par le VFJ et le SARS-CoV2 d’évolution fatale.

## Cas clinique

**Figure 1:**
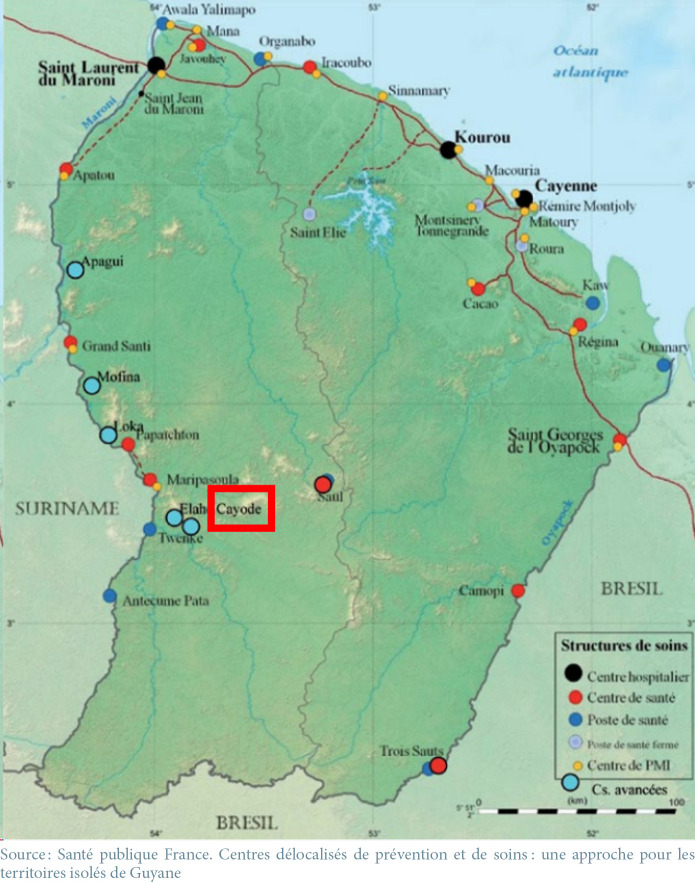
Structures de soins en Guyane

Le patient était un Amérindien de 14 ans né en Guyane [16]. Il vivait à Kayodé, village forestier du Haut-Maroni, en pays amérindien Wayana, à environ une heure et demie de pirogue de Maripasoula, ville la plus proche accessible par avion depuis Cayenne (Fig. 1). La famille, amérindienne teko, s’était installée dans le village quelques années plus tôt, arrivant de Camopi [6] (Fig. 1). Il présentait comme principal antécédent un retard de développement psychomoteur attribué à un syndrome d'alcoolisation fœtale. Il passait la majeure partie de son temps chez lui dans une maison en lisière de forêt, autonome dans la vie quotidienne, mais pas assez pour aller à l’école. Il n'avait pas de suivi médical régulier. Le 10 juillet, le patient a présenté de la fièvre, une rhinite, des céphalées, une toux, une asthénie et une anorexie, sans consulter de médecin. Le 6^e^ jour, devant un état de prostration, ses parents l'ont emmené au centre de dépistage, de prévention et de soins (CDPS) le plus proche, à Talhuen, à une demi-heure de pirogue de Kayodé (Fig. 1). Un état de coma fébrile a été constaté avec un score de Glasgow à 7/15, accompagné d'un syndrome méningé, la présence d'un signe de Babinski bilatéral et une héminégligence droite. Il a présenté par la suite deux crises d’épilepsie. L’évacuation médicale héliportée vers le service de réanimation au Centre hospitalier de Cayenne (CHC) n'a été possible que le lendemain.

Source : Santé publique France. Centres délocalisés de prévention et de soins : une approche pour les territoires isolés de Guyane

À l'arrivée en réanimation, le patient a présenté une polypnée à 35 cycles/min, une saturation à 100 % sous 9 l/min d'oxygène et une douleur de l'hypochondre droit. Il a été intubé, ventilé et traité par ceftriaxone 1 g/ jour dans l'hypothèse d'un sepsis grave et /ou d'une leptospirose et dexaméthasone 10 mg IV pour la Covid une fois par jour dès son arrivée (J7).

Tous les résultats biologiques à l'arrivée figurent dans le Tableau I. L'analyse des gaz du sang réalisés sous ventilation mécanique avec Fi à 40 % retrouvait une acidose métabolique d'origine lactique. Un tableau d'hépatite fulminante avec TP < 10 %, ALAT et ASAT supérieures au seuil maximal, et une insuffisance rénale aiguë KDIGO 3 à diurèse conservée était observé.

**Tableau I:** Caractéristiques biologiques du patient à l'admission en réanimation au CHC

Variable	Norme	Résultat
pH*	7,37-7,43	7,17
Pa (mmHg)*	75-100	81
pC (mmHg)*	35-45	44
HCO_3_^-^ (mmol/l)*	22-29	19
Lactates (mmol/l)	0,3-0,8	8,2
TP (%)	>70	<10
TCA	0,6-1,2	>6
ALAT (Ul/l)	<41	>7 000
ASAT (Ul/l)	<40	>7 000
Bilirubine totale/conjuguée (µmol/l)	<17 / 5	101,5 / 57,5
GGT (Ul/l)	<60	53
PAL (Ul/l)	<390	509
Hémoglobine (g/dl)	12,1-16,6	13,8
Plaquettes (G/l)	166-375	172
Leucocytes (G/l)	3,75-13	12,8
CRP (mg/l)	<5	63
Urée (mmol/l)	2,8-8,1	12,6
Créatinine (µmol/l)	40-68	326
Potassium (mmol/l)	3,5-5, 1	6
Sodium (mmol/l)	136-145	118
CPK (IU/l)	<195	3560
D-dimères (ng/ml)	<0,5	94 926
Fibrinogène (g/l)	2,38-4, 98	1,1
Lipase (UI/l)	13-60	982

* Gaz du sang réalisé sous ventilation mécanique Fi 40 %

Légende : ALAT : alanine aminotransférase; ASAT : aspartate aminotransferase; CPK : créatine phospho kinase; CRP : protéine C réactive; GGT : Gamma GT; HCO_3_^-^ : bicarbonates; PAL : phosphatases alcalines; Pa : pression artérielle en oxygène PC : pression artérielle en C02; TCA : temps de céphaline activée; TP : taux de prothrombine

Les explorations microbiologiques sont rapportées dans le Tableau II. La détection de l'antigène NS1 était positive pour le DENV, mais la confirmation par PCR était négative, suggérant un faux positif de l'AgNS1 lié à une réaction croisée entre *Orthoflavivirus.* Un sérum prélevé à J7 du début des symptômes s'est révélé fortement positif en PCR FJ avec la technique RT-qPCR de Weidmann *et al.* [24] et négatif en sérologie FJ (méthode Elisa) : absence de détection d'IgG ni d'IgM contre le virus de la FJ dans le sang à J7 du début des symptômes. L’écouvillon nasopharyngé était positif pour le SARS-CoV2 en PCR.

**Tableau II:** Examens microbiologiques réalisés à l'admission au CHC

Agent infectieux	Diagnostic direct	Sérologie
VIH	Ag P24 nég	
VHA		IgM nég - IgG pos
VHB	Ag HBs nég	Ac anti HBs nég
VHC		IgG nég
VHD		IgM nég
VHE		IgM nég
HHV8	CV ADN < 2 000 copies	
HHV6		IgG nég
CMV		IgM nég
EBV		IgM VCA nég - IgG anti-VCA et IgG anti-EBNA pos
Leptospirose	PCR nég sang et urines	IgM nég - IgG nég
Rubéole		IgM nég
HTLV		IgG nég
Dengue	Ag NS1 pos PCR nég	IgM nég
*S. pneumoniae*	Antigène urinaire nég	
*Legionella pneumophila*	Antigène urinaire néf	
SARS-CoV-2	PCR pos	
Rickettsioses		IgM nég - IgG nég
HSV1		IgM nég - IgG pos
HSV2		IgM nég - IgG nég
VZV		IgM nég - IgG pos
Coxsackie		IgM nég
Oreillons		IgM nég
PV B19		IgM nég
Rougeole		IgM nég
Paludisme	Frottis et goutte épaisse : nég	
Chagas		IgM nég
Toxoplasmose	PCR nég	IgM nég - IgG nég
*C. pneumoniae*		IgM nég - IgG nég
*M. pneumoniae*		IgM nég - IgG nég
Fièvre Jaune	PCR pos	IgM nég - IgG nég
Tonate		IgM nég
Mayaro		IgM nég
Chikungunya		IgM nég
Hémocultures	Culture négative	
LCR	Culture négative	
ECBU	Culture négative	

Légende : Ac : anticorps; Ag : antigène; CMV : cytomégalovirus; EBNA : Epstein-Barr Nuclear Antigen; EBV : Epstein Barr virus; ECBU : examen cyto bactériologique des urines; HBc : core du virus de l'hépatite B; HBs : surface du virus de l'hépatite B; HHV6 : virus herpétique humain 6; HHV8 : virus herpétique humain 8; HSV1 : virus Herpès simplex de type 1; HSV2 : virus Herpès simplex de type 2; HTLV : virus T-lymphotrope humain; LCR : liquide céphalo rachidien; Nég : négatif; Pos : positif; PV B19 : Parvovirus B19; SARS-CoV-2 : coronavirus 2 du syndrome respiratoire aigu sévère; VCA : Viral Capsid Antigen; VHA : virus de l'hépatite A; VHB : virus de l'hépatite B; VHC : virus de l'hépatite C; VHD : virus de l'hépatite D; VHE : virus de l'hépatite E; VIH : Virus d'immunodéficience humaine; VZV : varicella zona virus

Le scanner cérébral a montré un effacement des citernes de la base au niveau du trou occipital. Le scanner thoraco-abdomino-pelvien avec injection de produit de contraste a retrouvé une hypoventilation distale des deux pyramides basales réalisant des comblements alvéolaires, et un épanchement péritonéal et rétropéritonéal diffus de moyenne abondance. Il n'a pas montré de signe évocateur d'infection pulmonaire à SARS-CoV2.

Rapidement après l'admission on constate l'aggravation de la défaillance multiviscérale, avec une défaillance hémodynamique nécessitant de la noradrénaline jusqu’à 20 mg/h, une défaillance neurologique avec un coma, des crises d’épilepsie et un myosis serré bilatéral évoluant progressivement vers une mydriase bilatérale, une défaillance respiratoire avec augmentation progressive des besoins en oxygène sous ventilation mécanique (Fi 100 %) et enfin une défaillance rénale score KDIGO 3 (Kidney Disease/Improving Global Outcomes) et hépatique. L’épuration extra-rénale a été initiée avec une mauvaise tolérance hémodynamique. Une évacuation médicale vers un centre de transplantation hépatique en France métropolitaine a été envisagée, mais récusée en raison de l'instabilité hémodynamique du patient, non compatible avec un transport en avion de plusieurs heures, et de sa contagiosité par le SARS-CoV2 dans l'avion de ligne qui réalise les évacuations vers l'hexagone. L'adolescent est décédé au 9^e^ jour de l'apparition des premiers symptômes (2^e^ jour d'hospitalisation en réanimation).

L'enquête menée à l'issue de l'hospitalisation a révélé que le patient a reçu une première dose de vaccin contre la FJ avant l’âge de deux ans à Camopi, mais qu'il n'a pas reçu le rappel habituellement recommandé à partir de six ans en Guyane. En raison de l'absence de détection d'IgG anti-VFJ, et en l'absence de réalisation d'une recherche d'anticorps neutralisants, il est difficile de savoir si le patient n’était pas protégé contre le VFJ ou s'il disposait encore d'anticorps neutralisants à titre théoriquement protecteur (échec vaccinal vrai).

## Discussion

À notre connaissance, il s'agit du premier cas de co-infection SARS CoV2-VFJ rapporté dans la littérature. Les co-infections avec le SARS-CoV2 les plus fréquentes chez les patients hospitalisés concernent les bactéries pour 7 % [11]; seulement 3 % concernent des virus, principalement le VRS et la grippe A [21]. Dans les zones tropicales, des co-infections ont déjà été décrites avec un autre *Orthoflavivirus,* le DENV (virus de la dengue) comme cela avait été rapporté à Mayotte et à la Réunion au début de l’épidémie de dengue DENV1 en mars 2020 [7, 23]. Une co-infection fièvre jaune et virus de l'encéphalite japonaise a également été rapportée en Angola en mars 2016 [18].

Il est difficile de savoir si la co-infection par le SARS-CoV2 a aggravé le tableau clinique de la FJ, cette dernière étant habituellement suffisamment sévère pour l'expliquer. En outre, c'est l'atteinte respiratoire qui fait la gravité de la Covid-19 et dans le cas de ce patient, le scanner n'a pas retrouvé les lésions habituellement associées aux formes pulmonaires graves, malgré une défaillance respiratoire clinique.

Par ailleurs, la situation sanitaire liée à la pandémie de Covid-19 a probablement contribué à la difficulté de la prise en charge médicale [14]. Deux éléments ont empêché l’évacuation sanitaire vers un centre spécialisé en France hexagonale pour une transplantation hépatique : tout d'abord, l’état clinique du patient s'est dégradé trop rapidement pour qu'un transfert aussi long par avion soit possible; ensuite, la positivité de ses échantillons respiratoires au SARS-CoV2, qui contre-indiquait à l’époque la possibilité d'un transfert par un avion de ligne en raison du risque de contamination des autres passagers. La transplantation hépatique, qui n'aurait été possible qu'en métropole, ne permet pas toujours de sauver les patients. En 2020, un patient transporté en urgence en France hexagonale pour transplantation hépatique sur hépatite fulminante liée à une fièvre jaune est décédé en post- opératoire [16]. Au cours de l’épidémie brésilienne de 2018, un total de sept patients avaient pu bénéficier d'une transplantation hépatique parmi lesquels seulement trois avaient survécu [5].

En outre, d'autres éléments que la Covid-19 ont pu contribuer à cette difficulté de prise en charge : les conditions socio-économiques précaires, l'absence de scolarisation, le retard de recours aux soins, la mise sous corticoïdes devant une gravité initiale attribuée à la Covid, le handicap du patient et enfin l'isolement sanitaire des habitants de Kayodé.

Concernant l’état vaccinal du patient, la recherche des anticorps neutralisants de la fièvre jaune par une technique de neutralisation (type PRNT80 ou 90) n'a pas été réalisée, ne permettant pas de conclure si cette absence d'IgG anti-fièvre jaune était liée à un échec vaccinal ou à une non-persistance à long terme des anticorps antiamarils. Mais la priorisation du prélèvement s'est portée sur l'isolement viral dans ce contexte de suspicion clinique de fièvre jaune aiguë.

Une enquête épidémiologique a été menée un mois plus tard, avec vérification et correction du statut vaccinal de l'ensemble de la population locale de Kayodé (Clara Pichard, Céline Michaud, Lise Dudognon et Mélanie Gaillet, données non publiées). Les statuts vaccinaux de 287 résidents du village de Kayodé, parmi lesquels 102 enfants de moins de 14 ans, ont pu être évalués : 4 (1,4 %) étaient en retard pour la 1^re^ dose de vaccin fièvre jaune, 36 (12,5 %) pour la deuxième dose et 247 personnes étaient à jour de leur vaccination fièvre jaune soit 86,1 % de la population du village. Des mesures de lutte antivectorielle ont été rapidement mises en place dans le village par le service de Lutte antivectorielle de la Collectivité territoriale de Guyane : suppression des eaux stagnantes au domicile et dans les alentours (gouttières, vases, pneus usagés, soucoupes de pots à plantes, tout autre récipient pouvant contenir de l'eau stagnante); évacuation de l'eau des bâches; tontes régulières des jardins.

En Guyane, la couverture vaccinale contre le VFJ était estimée à 95 % en 2017, avec des chiffres un peu moins élevés le long du fleuve Maroni : 62,3 % à Grand-Santi, 76,9 % à Saint Laurent du Maroni et 78,3 % à Papaïchton [9]. Deux doses de vaccins contre la FJ sont recommandées dans l'enfance, l'une entre 9 mois et 2 ans et la seconde entre 6 et 12 ans [13]. Cette recommandation n'est cependant pas homogène sur toute la région amazonienne : elle n'est pas appliquée dans certains pays comme le Venezuela où une seule dose de vaccin dès l’âge de neuf mois suffit [21], alors qu'une recommandation équivalente existe au Brésil, qui a introduit une dose de rappel à 4 ans en 2020 [12]. Ce cas suggère que cette dose de rappel vaccinal antiamaril devrait être administrée systématiquement. C'est ce que confirme une métanalyse publiée en janvier 2024 dans le Lancet Global Health : ces populations de moins de deux ans, vivant en zone d'endémie, sont plus à risque [17]. Chez l'adulte aussi il convient d’être prudent en affirmant qu'une seule injection de vaccin contre le VFJ est suffisante pour la vie [2]. L'impact de la pandémie de Covid-19 a pu être important sur la couverture vaccinale des patients, en particulier sur les rappels de vaccins dans la population générale et pédiatrique. Un retard de 6 à 8 mois à l'issue de l’épidémie de Covid, lié à la réorganisation nécessaire des soins a été mis en évidence à Kayodé en août 2020, concernant la deuxième dose de vaccination contre le VFJ par le service de Protection maternelle et infantile [1]. Par ailleurs, la Guyane a connu à compter de 2021 une vague massive d'hésitation vaccinale liée à la vaccination anti-Covid-19 [4], qui s'est progressivement étendue à d'autres vaccins, ce qui risque de compliquer les futures campagnes de vaccination. Nous craignons que des retards similaires se soient produits dans d'autres pays d'Amérique du Sud, ce qui pourrait favoriser l'apparition de nouvelles épidémies de FJ.

## Conclusion

Nous avons rapporté un cas exceptionnel de coinfection VFJ - SARS-CoV2, dont l'issue a été fatale. Le tableau clinique était principalement celui d'une hépatite fulminante, probablement liée au VFJ, avec une atteinte respiratoire clinique sévère, sans image radiologique évocatrice d'infection à SARS-CoV2. La co-infection par la Covid-19 a limité la possibilité de transfert du patient vers un centre de transplantation hépatique, déjà compromis par l'aggravation rapide de son état clinique. Ce cas a été l'occasion de mettre en évidence les couvertures vaccinales insuffisantes dans les territoires isolés en Guyane. Les programmes vaccinaux ont été renforcés par la suite. L'hésitation vaccinale globale, qui a suivi celle contre la Covid-19, risque d'avoir un retentissement sur la couverture des autres vaccins recommandés.

## Financement

Ce travail n'a bénéficié d'aucune source de financement.

## Consentement du patient

Le patient n'a pas manifesté d'opposition de son vivant à l'utilisation de ses données à des fins de recherche.

## Contribution des auteurs

La rédaction du cas a été réalisée par l'auteur principal, aidé du dernier auteur. Tous les coauteurs ont émis des suggestions pertinentes pour l'aide à la rédaction de ce cas, et ce de manière égale.

## Déclaration d'intérêt

Les auteurs ne rapportent aucun conflit d'intérêt.
