# Supplementary material for: Fatal case of co-infection with yellow fever virus and SARS-CoV2 during the 2020 Covid-19 pandemic in French Guiana
Source: Med Trop Sante Int. 2024 Sep 12;4(3):mtsi.v4i3.2024.445. [Article in French] doi: 10.48327/mtsi.v4i3.2024.445 (PMC11809067; doi:10.48327/mtsi.v4i3.2024.445)
Supplement: Related texts

# Research article: Fatal case of co-infection with yellow fever virus and SARS-CoV2 during the 2020 Covid-19 pandemic in French Guiana

## Introduction

Yellow fever virus (YFV), recently renamed *Orthoflavivirus flavi,* is an arbovirus of the Flaviviridae family and the *Orthoflavivirus* genus^1^ [3]. It is an endemic arbovirosis, the most serious circulating in Latin America. In 2016-2018, an epidemic occurred in Brazil, affecting areas previously free of the disease [15]. In the Guiana Shield, which includes the Venezuelan state of Bolivar, Guyana, Surinam, French Guiana and the Brazilian state of Amapá, only nine cases were reported between 1990 and 2022 [22]. In December 2019, the global Covid-19 pandemic began and quickly reached South America. The first cases were reported in French Guiana in March 2020 [8]. The possibility of co-infection is high in this zone, where numerous infectious agents circulate [6]. Indeed, in July 2020, French Guiana experienced its first wave of Covid-19 as well as an epidemic of dengue fever, mainly due to serotype DENV2 [10]. Sporadic cases of malaria, mainly *Plasmodium vivax,* also occur regular on the Haut-Maroni [20]. In addition, leptospirosis remains prevalent, increasing the number of differential diagnoses. We report the case of a patient with fatal co-infection with YFV and SARS-CoV2.

## Clinical case

The patient was a 14-year-old Amerindian boy born in French Guiana [16]. He lived in Kayodé, a forested village on the Haut-Maroni in Wayana Amerindian country, about an hour and a half by pirogue from Maripasoula, the nearest town accessible by air from Cayenne (Fig. 1). The family, Teko Amerindians, had settled in the village a few years earlier, coming from Camopi [6] (Fig. 1). His main antecedent was delayed psychomotor development attributed to fetal alcohol syndrome. He spent most of his time at home in a house at the edge of the forest, independent in daily life, but not enough to attend school. He had no regular medical follow-up.

**Figure 1:** Health structure in French Guiana

Source : Santé publique France. Centres délocalisés de prévention et de soins : une approche pour les territoires isolés de Guyane

On July 10, the patient presented fever, rhinitis, headache, cough, asthenia and anorexia, without consulting a doctor. On the 6th day, faced with a state of prostration, his parents took him to the nearest screening, prevention and care center in Talhuen, half an hour by pirogue from Kayodé (Fig. 1). He was in a febrile coma with a Glasgow score of 7/15, accompanied by meningeal syndrome, bilateral Babinski sign and right hemineglect. He subsequently had two epileptic seizures. Medical evacuation by helicopter to the intensive care unit (ICU) of the Centre hospitalier de Cayenne (CHC) was not possible until the following day.

On arrival to the ICU, the patient presented with polypnoea at 35 cycles/min, 100% saturation on oxygen with 9 L/min and right hypochondrium pain. He was intubated, ventilated and treated with ceftriaxone 1 g/day in the hypothesis of severe sepsis and/or leptospirosis and dexamethasone 10 mg IV for Covid once daily on arrival (D7).

All biological results on arrival are shown in Table I. Blood gas analysis under mechanical ventilation with Fi at 40% showed metabolic acidosis of lactic origin. Fulminant hepatitis with PT < 10%, ALAT and ASAT above the maximum threshold, and acute renal failure KDIGO 3 with preserved diuresis was observed.

**Table I:** Biological characteristics of the patient at admission to the CHC intensive care unit

Variable	Standard	Result
pH*	7,37-7,43	7,17
Pa (mmHg)*	75-100	81
pC (mmHg)*	35-45	44
HCO_3_^-^ (mmol/L)*	22-29	19
Lactate (mmol/L)	0,3-0,8	8,2
PT (%)	>70	<10
aPTT	0,6-1,2	>6
ALAT (IU/L)	<41	>7 000
ASAT (IU/L)	<40	>7 000
Total/conjugated bilirubin (µmol/L)	<17 / 5	101,5 / 57,5
GGT (IU/L)	<60	53
ALP (IU/L)	<390	509
Hemoglobin (g/dL)	12,1-16,6	13,8
Platelets (G/L)	166-375	172
Leukocytes (G/L)	3,75-13	12,8
CRP (mg/L)	<5	63
Urea (mmol/L)	2,8-8,1	12,6
Creatinine (µmol/L)	40-68	326
Potassium (µmol/L)	3,5-5, 1	6
Sodium (mmol/L)	136-145	118
CPK (IU/L)	<195	3560
D-dimers (ng/mL)	<0,5	94 926
Fibrinogen (g/L)	2,38-4, 98	1,1
Lipase (IU/L)	13-60	982

* Blood gas under mechanical ventilation Fi 40

Legend: ALAT: alanine aminotransferase; ALP: alkaline phosphatase; aPTT: activated partial thrombosis time; ASAT: aspartate aminotransferase; CPK: creatine phospho kinase; CRP: C-reactive protein; GGT:Gamma-GT; HCO_3_^-^: bicarbonates; Pa: blood pressure in oxygen PC: blood pressure in C; PT : Prothrombin Levels

Microbiologic studies are reported in Table II. NS1 antigen detection was positive for DENV, but PCR confirmation was negative, suggesting a false positive for AgNS1 related to cross-reactivity between orthoflaviviruses. A serum collected at D7 of symptom onset was highly positive for YF PCR using the RT-qPCR technique of Weidmann *et al.* [24] and negative for YF serology (Elisa method): neither IgG nor IgM against YF virus was detected in the blood at D7 of symptom onset. The nasopharyngeal swab was PCR positive for SARS-CoV2.

**Table II:** Microbiological examinations carried out on admission in he CHC

Infectious agent	Direct diagnosis	Serology
HIV	P24 Ag neg	
HAV		IgM neg - IgG pos
HBV	HBs Ag neg	Anti HBs Ab neg
HCV		IgG neg
HDV		IgM neg
HEV		IgM neg
HHV8	ml	
HHV6		IgG neg
CMV		IgM neg - IgG neg
EBV		VCA IgM neg - anti-VCA IgG and anti-EBNA IgG: Pos
Leptospirosis	Blood and urine PCR neg	IgM neg - IgG neg
Rubella		IgM neg
PTLVs		IgG neg
Dengue fever	NS1 Ag pos PCR neg	IgM neg
*S. pneumoniae*	Urinary antigen neg	
*Legionella pneumophila*	Urinary antigen neg	
SARS-CoV-2	PCR pos	
Rickettsiosis		IgM neg - IgG neg
HSV1		IgM neg - IgG pos
HSV2		IgM neg - IgG neg
VZV		IgM neg - IgG pos
Coxsackievirus		IgM neg
Mumps		IgM neg
PV B19		IgM neg
Measles		IgM neg
Malaria	Smear and thick drop: neg	
Malaria		IgM neg
Malaria	PCR neg	IgM neg - IgG neg
*C. pneumoniae*		IgM neg - IgG neg
*M. pneumoniae*		IgM neg - IgG neg
Yellow fever	PCR pos	IgM neg - IgG neg
Tonate		IgM neg
Mayaro		IgM neg
Chikungunya		IgM neg
Blood cultures	Negative culture	
CSF	Negative culture	
CBEU	Negative culture	

Legend: Ag: antigen; CBEU: cytobacteriological examination of urine; CMV: Cytomegalovirus; CSF : cerebrospinal fluid; EBNA: Epstein-Barr Nuclear Antigen; EBV: Epstein Barr virus; CBEU: Cytobacteriological examination of urine; HBc: hepatitis B virus core; HBs: surface of the hepatitis B virus; HHV6: Human Herpes Virus 6; HHV8: Human Herpes Virus 6; HSV1: herpes simplex virus 1; HSV2: herpes simplex virus 2; HTLV: Human T-lymphotropic virus; CSF: cerebrospinal fluid; mL: mm; Neg: negative; Pos: positive; PV B19: Parvovirus B19; VCA: Viral Capsid Antigen; HAV: hepatitis A virus; HBV: hepatitis B virus; HCV: hepatitis C virus; HDV: hepatitis D virus; HEV: hepatitis E virus; HIV: Human Immunodeficiency Virus; SARS-CoV-2 : Severe Acute Respiratory Syndrome Coronavirus 2; VZV: varicella zoster virus

Cerebral CT scan showed effacement of the basal cisterns at the level of the foramen magnum. A thoraco-abdomino-pelvic scan with contrast agent showed distal hypoventilation of the two basal pyramids, with alveolar filling, and a diffuse, moderately abundant peritoneal and retroperitoneal effusion. He did not have any signs suggestive of SARS-CoV2 pulmonary infection. Multivisceral failure rapidly worsened after admission, with hemodynamic failure requiring norepinephrine up to 20 mg/h, neurological failure with coma, seizures, and bilateral tight miosis progressing to bilateral mydriasis, respiratory failure with progressive increase in oxygen requirements on mechanical ventilation (Fi 100%), and finally renal failure with KDIGO 3 (Kidney Disease/Improving Global Outcomes) and hepatic failure. Extra-renal dialysis was initiated due to poor hemodynamic tolerance. Medical evacuation to a liver transplant center in mainland France was considered, but was denied due to the patient's hemodynamic instability, which was incompatible with several hours of air transport, and his contagiousness with SARS-CoV2 in the passenger aircraft used for evacuation. The teenager died on the 9th day after the onset of symptoms (2nd day in intensive care).

Investigation after hospitalization revealed that the patient had received a first dose of yellow fever vaccine in Camopi before the age of 2, but had not received the booster vaccine usually recommended for six-year-olds in French Guiana. In the absence of anti- YFV IgG detection and neutralizing antibody testing, it is difficult to know whether the patient was unprotected against YFV or still had neutralizing antibodies at a sufficient level for theoretical protection (true vaccine failure).

## Discussion

To our knowledge, this is the first case of SARSCoV2-YF co-infection reported in the literature. The most common co-infections with SARS-CoV2 in hospitalized patients are bacterial (7% [11]); only 3% are viral, mainly RSV and influenza A [21]. In tropical areas, co-infections with another orthoflavivirus, DENV (dengue virus) have been described, as reported in Mayotte and Réunion at the beginning of the DENV 1 epidemic in March 2020 [7, 23]. Co-infection with yellow fever and Japanese encephalitis virus was also reported in Angola in March 2016 [17].

It is unclear whether co-infection with SARSCoV2 aggravated the clinical picture of YF, as the latter is usually severe enough to explain it. Furthermore, the severity of Covid-19 is determined by respiratory involvement, and, in the case of this patient, despite clinical respiratory failure, the CT scan did not show the lesions usually associated with severe pulmonary forms. In addition, the health situation associated with the Covid-19 pandemic probably contributed to the difficulty of medical management [14]. Two factors prevented medical evacuation to a specialized center in mainland France for liver transplantation: first, the patient's clinical condition deteriorated too rapidly for such a long transfer by air; second, his respiratory samples were positive for SARS-CoV2, which at the time contraindicated the possibility of air transfer due to the risk of contamination of other passengers. Liver transplantation, which would have been available only in mainland France, does not always save patients. In 2020, a patient transported to mainland France for liver transplantation for fulminant hepatitis associated with yellow fever died postoperatively [16]. During the 2018 epidemic in Brazil, a total of seven patients received liver transplants, only three of whom survived [5]. In addition to Covid-19, other factors may have contributed to the difficulty in managing the medical care: precarious socioeconomic conditions, lack of education, delayed access to care, the initial severity of the patient's condition attributed to Covid, the need to take corticosteroids, the patient's disability and, last but not least, the health isolation of the people of Kayodé.

With regard to the patient's vaccination status, the yellow fever neutralizing antibody test (PRNT80 or 90 type) was not performed, making it impossible to conclude whether the absence of yellow fever IgG was related to vaccine failure or longterm non-persistence of yellow fever antibodies. However, in this context of clinical suspicion of acute yellow fever, priority was given to virus isolation.

One month later, an epidemiological survey was conducted to verify and correct the vaccination status of the entire local population of Kayodé (Clara Pichard, Céline Michaud, Lise Dudognon and Mélanie Gaillet, unpublished data). The vaccination status of 287 inhabitants of the village of Kayodé, including 102 children under 14 years of age, was assessed: 4 (1.4%) were overdue for the 1st dose of yellow fever vaccine, 36 (12.5%) for the second dose, and 247 people (86.1% of the village population) were up to date with their yellow fever vaccination. Vector control measures were quickly implemented in the village by the Vector Control Department of French Guiana: removal of standing water from homes and surrounding areas (gutters, vases, used tires, plant pot saucers, and any other containers that may contain standing water); drainage of water from tarps; regular mowing of gardens.

In French Guiana, YF vaccination coverage was estimated at 95% in 2017, with slightly lower figures along the Maroni River: 62.3% in Grand-Santi, 76.9% in Saint Laurent du Maroni and 78.3% in Papaïchton [9]. Two doses of YF vaccine are recommended in childhood, one between 9 months and 2 years of age and the second between 6 and 12 years of age [13]. However, this recommendation is not uniform throughout the Amazon region: it is not applied in some countries, such as Venezuela, where a single dose of vaccine at 9 months of age is sufficient [21], whereas an equivalent recommendation exists in Brazil, which introduced a booster dose at 4 years of age in 2020 [12]. This case suggests that this yellow fever booster dose should be administered systematically. This is confirmed by a meta-analysis published in Lancet Global Health in January 2024: these populations under 2 years of age living in endemic areas are at higher risk [17]. Even in adults, we must be cautious in claiming that a single injection of YF vaccine is sufficient for life [2].

The impact of the Covid-19 pandemic may have been significant on the vaccination coverage of patients, particularly on booster doses in the general and pediatric populations. A delay of 6 to 8 months at the end of the Covid epidemic, related to the necessary reorganization of care, was highlighted in Kayodé in August 2020, for the second dose of YF vaccination by the Maternal and Child Protection Service [1]. In addition, French Guiana experienced a massive wave of vaccine hesitancy in 2021 related to Covid-19 vaccination [4], which gradually spread to other vaccines, potentially complicating future vaccination campaigns. We fear that similar delays have occurred in other South American countries, which could facilitate the emergence of new YF epidemics.

## Conclusion

We report an exceptional case of YF-SARS-CoV2 co-infection, with a fatal outcome. The clinical picture was predominantly a fulminant hepatitis, probably related to YF, with severe clinical respiratory involvement, without radiological findings suggestive of SARS-CoV2 infection. Coinfection with Covid-19 limited the possibility of transferring the patient to a liver transplant center, already compromised by the rapid deterioration of his clinical condition. This case highlighted the lack of efficient vaccination coverage in isolated areas of French Guiana. Vaccination programs were subsequently intensified. The global vaccine hesitancy that followed the Covid-19 case is likely to affect coverage of other recommended vaccines.

## Funding

No funding was provided for this work.

## Patient's consent

The patient has not objected to the use of his data for research purposes during his lifetime.

## Authors’ contributions

The case was written by the lead author with the assistance of the last author. All co-authors made relevant suggestions to help write the case and contributed equally.

## Conflict of interest

The authors declare no conflict of interests.
